# Comparisons of gene coexpression network modules in breast cancer and ovarian cancer

**DOI:** 10.1186/s12918-018-0530-9

**Published:** 2018-04-11

**Authors:** Shuqin Zhang

**Affiliations:** Center for Computational Systems Biology, Shanghai Key Laboratory for Contemporary Applied Mathematics, School of Mathematical Sciences, Fudan University, No.220 Handan Road, Shanghai, 200433 China

**Keywords:** breast cancer, Ovarian cancer, Module comparison

## Abstract

**Background:**

Breast cancer and ovarian cancer are hormone driven and are known to have some predisposition genes in common such as the two well known cancer genes BRCA1 and BRCA2. The objective of this study is to compare the coexpression network modules of both cancers, so as to infer the potential cancer-related modules.

**Methods:**

We applied the eigen-decomposition to the matrix that integrates the gene coexpression networks of both breast cancer and ovarian cancer. With hierarchical clustering of the related eigenvectors, we obtained the network modules of both cancers simultaneously. Enrichment analysis on Gene Ontology (GO), KEGG pathway, Disease Ontology (DO), and Gene Set Enrichment Analysis (GSEA) in the identified modules was performed.

**Results:**

We identified 43 modules that are enriched by at least one of the four types of enrichments. 31, 25, and 18 modules are enriched by GO terms, KEGG pathways, and DO terms, respectively. The structure of 29 modules in both cancers is significantly different with *p*-values less than 0.05, of which 25 modules have larger densities in ovarian cancer. One module was found to be significantly enriched by the terms related to breast cancer from GO, KEGG and DO enrichment. One module was found to be significantly enriched by ovarian cancer related terms.

**Conclusion:**

Breast cancer and ovarian cancer share some common properties on the module level. Integration of both cancers helps identifying the potential cancer associated modules.

**Electronic supplementary material:**

The online version of this article (10.1186/s12918-018-0530-9) contains supplementary material, which is available to authorized users.

## Background

Despite of decades of intensive study and substantial progress in understanding breast cancer and ovarian cancer, these two diseases remain the most prevalent malignancy in women, and the important causes of death in women. Among solid gynaecological tumors, breast cancer is the most often diagnosed tumor while ovarian cancer is the most deadly gynaecological neoplasia. Both breast and ovarian cancer are hormone driven and are known to have some predisposition genes in common. The major genes associated with susceptibility to breast and ovarian cancer are the two well-known high-penetrance cancer genes: BRCA1 and BRCA2 [1–3]. However, mutations in these genes account for only a very small percent of all breast cancers and ovarian cancers. Other genes such as TP53, PTEN, and STK11/LKB1, are even less common causes of breast and ovarian cancer [4]. Despite tremendous efforts to conquer such malignant diseases, research on studying the mechanism of cancer development and developing effective preventive measures is still a hot topic.

The high speed development of high-throughput technologies such as next generation sequencing of the human genome, gene expression microarrays, identification of the changes of copy numbers has dramatically accelerated the study aiming at predicting and curing such diseases. Many works have been published to address the topics on associated susceptibilities, potential biomarkers, cancer predictions and so on [5–19]. Several of them put breast cancer and ovarian cancer together in their studies [5–10]. These works either borrowed information from each other with the assumption that both cancers have similar etiologies [5, 7, 9], or conducted research on the differences between the pathogenic mechanisms [6, 8]. Some review papers also analyzed the related research progresses of both cancers together [4, 10]. In this work, we also put both cancers together to study the complex gene coexpression patterns with the network tools.

Network has been widely applied to study the complex interactions between genes, proteins, and other small molecules. It is also a popular tool for studying breast cancers and ovarian cancers [11–16, 20–22]. When using networks to study the complex interactions, one typical concept is module, which is the densely connected subnetworks. With the network module analysis, we may infer the susceptibility genes in cancer [16], identify the biomarkers [14], and predict the prognosis of the cancer patients [11]. Current studies on cancer-related network modules are mainly based on one network, which is also true for breast cancer [14] and ovarian cancer [16]. Even in the study of more than one network, the module identification process is one network by one network, and then comparisons between different networks are performed [16]. Recently, several module identification methods on multiple networks are proposed [22–24]. Among them, the method proposed in [22] introduced an algorithm to find the differential modules in different networks. It mainly concentrates on the differential part. While in the paper [24], the method not only can find the modules in each network, but also can align the modules at the same time. Thus both the common and the differential parts can be detected. In the following, we compared the modules that were identified from the gene coexpression networks of breast cancer and ovarian cancer using the method in [24]. We analyzed the basic properties of the modules including density, average degree, distribution difference etc., and we did enrichment analysis of Gene Ontology (GO), KEGG pathway, Disease Ontology (DO) and Gene Set Enrichment Analysis (GSEA). By comparing the modules, both the common properties and the differences between the two cancers are detected.

## Methods

### Data sets

The level 3 gene expression data for breast cancer (BRCA) and ovarian cancer (OV) were downloaded from The Cancer Genome Atlas (TCGA). The gene expression data were generated with UNC AgilentG4502A. We chose the samples from the solid tissues only. There are 526 and 572 samples for both BRCA and OV, respectively. The expression value of 17,814 genes was measured. The missing data for each specific gene was imputed with the mean value of the known samples. We also downloaded the most updated protein-protein interaction (PPI) data for humans from BioGrid *https://thebiogrid.org*. We chose the genes having PPIs in the gene expression data, and 9603 genes were selected.

To choose the differential expressed genes for BRCA and OV, *t*-test and Kolmogorov-Smirnov test were applied. The genes having *p*-values less 0.01 in both tests were chosen, where the *p*-values were adjusted by controlling the false discovery rate. We then got 7742 genes.

### Coexpression network construction

We first computed the pairwise Pearson correlation coefficient *r*_*ij*_ to measure the coexpression levels between gene *i* and gene *j*. To make the correlations across the two networks comparable, we ‘normalized’ the Pearson correlation coefficients with the same method as shown in [25]. Fisher’s *z* transformation score for each *r*_*ij*_ was first computed as $z_{ij}=0.5\log \frac {1+r_{ij}}{1-r_{ij}}$. The *z*-scores were then normalized to make *z*_*ij*_ follow normal distribution. After this, the ‘normalized’ correlations were obtained by transforming back to *r*_*ij*_. With these steps, the correlations in the two networks will be on the same level. We then did hard thresholding to make the network be unweighted. We took 99.5% quantile of the transformed correlation coefficients as the threshold. If the absolute value of the correlation coefficient is greater than the threshold, we assigned an edge between the corresponding genes, otherwise, there is no edge. With this method, the average degree is about 37 in both networks.

### Module identification in BRCA and OV coexpression networks

Before we did module identification, we first removed the genes having no links with any other genes in both networks. The left gene number became 6779. Consider the constructed gene coexpression network *G*_1_ (BRCA), *G*_2_ (OV) consisting of 6779 genes. We let the adjacency matrices for both networks be *A*_1_,*A*_2_, where *A*_*k*_(*i*,*j*)=1 represents there is an edge between gene *i* and gene *j*. We first applied the model proposed in [24] to cluster the genes in lower subspace for both networks. This model aims at finding the clusters in multiple networks and aligning the clusters at the same time. The main idea is to use spectral clustering to find the cluster in each network, and align them by maximizing the cluster similarity of multiple networks. Here, we only have two networks. We assume the putative number of clusters *M* in both networks is given first.

Let *S*^*k*^ be the assignment of the 6779 vertices into *M* clusters for the network *G*_*k*_, where ${S}_{im}^{k}=1$, if *i*∈*G*_*k*_ belongs to the *m*-th cluster, otherwise ${S}_{im}^{k}=0$ for *i*=1,2,⋯,6779,*m*=1,2,⋯,*M*,*k*=1,2. The optimization model is formulated as:

1 $$\begin{array}{@{}rcl@{}} \min &&\,\, \sum\limits_{k=1,2}\sum\limits_{m=1}^{M}\frac{{S_{,m}^{k}}^{T}(D_{k}-A_{k}){S}_{,m}^{k}}{{S_{,m}^{k}}^{T}{S}_{,m}^{k}}-\beta\sum\limits_{k,l=1,2}\sum\limits_{m=1}^{M}\frac{{S_{,m}^{k}}^{T}S_{,m}^{l}}{\|S_{,m}^{k}\|_{2}\|S_{,m}^{l}\|_{2}}\\ \text{s.t.} &&\,\, S_{i,m}^{k}\in \{0,1\},i=1,2,\cdots,6779; m=1,2,\cdots,M; k=1,2;\\ &&\,\,\sum\limits_{m=1}^{M}{S}_{,m}^{k}=\mathbf{1},\text{for}\,\,k=1,2. \end{array} $$

The first term in the objective function is to do clustering in both networks separately, and the second term defines the similarity of the clusters in both networks measured by cosine function. *β* balances the contributions from inter-network and intra-network.

To solve the optimization problem (1), the variable $S^{k}_{im}$ is relaxed. Using the same technique as spectral clustering, the optimization problem is transformed to:

$$\begin{array}{@{}rcl@{}} \min \,\, {\tilde\Psi}(\tilde S)=\text{Tr}(\tilde S^{T}C\tilde S),\,\text{s.t.} \,\, \tilde S^{T}\tilde S=2I_{M}, \end{array} $$

where $ C=\left (\begin {array}{cc} L_{1}&{}\mathbf {0}\\ \mathbf {0}&L_{2}\\ \end {array} \right) -\beta \left (\begin {array}{cccc} \mathbf {0}&I_{n}\\ I_{n}&0\\ \end {array} \right), \tilde S = \left (\begin {array}{c} \tilde S^{1}\\ \tilde S^{2}\\ \end {array} \right), $$\tilde S_{,m}^{k}=\frac {S_{,m}^{k}}{\|S_{,m}^{k}\|_{2}}$, and *L*_*k*_=*D*_*k*_−*A*_*k*_, **0** is an *n*×*n* matrix with all entries being zero.

By computing the eigenvectors corresponding to the *M* smallest eigenvalues of matrix *C*, the original vertices in the networks are projected to a space of dimension *M*. To get the clusters, we may use *k*-means clustering to cluster the data points similar to spectral clustering. Due to the large size of the network, *k*-means does not work well. Instead, we applied hierarchical clustering with complete linkage to cluster the vertices. The distance is chosen to be the spearman distance. This is because when the size of matrix *C* is large, the range of the eigenvector entries is large, but their order is comparatively stable. The algorithm is summarized in ‘**Algorithm**’.



*Selection of parameter β and M* The parameter *β* controls the connections between the vertices in both networks. When *β*=0, it is equivalent to finding the clusters in two networks separately. When *β* becomes larger, the corresponding vertices in both networks tend to belong to the same cluster. We note that even when a group of vertices are densely connected in the first network, while their corresponding parts are isolated in the second network, the method will put all the isolated vertices in the same cluster as in the first network. Here, since both networks were controlled to have a close number of total connections, we directly set *β*=1, which means the connection weight between two networks is the same as that within both networks. We note that when *β*>1 and it is within a reasonable range, the results do not change much.

The number of clusters *M* was chosen according to the eigenvalues of matrix *C*. *M* corresponds to the first big eigengap [26]. We note that here *M* is not the number of clusters in either of the two networks because by choosing *β*, the isolated vertices in one network can also be clustered together depending on the other network. It should be the number of the union clusters in both networks. Thus the method can find both the consistent clusters and the differential clusters having quite different connection probabilities.

After the above clustering procedures, we can get a cluster label for each gene. The corresponding clusters in both networks may include different genes. We take the union of the genes as the cluster. Due to the large number of genes, the clustering method may have some bias. Some unconnected subnetworks may be clustered together. Before going to further analysis, we need to check each cluster such that it cannot include unconnected subnetworks. These resulted subnetworks are defined as the modules we identified.

### Enrichment analysis

To see the associations between the identified gene modules and gene functions, we did Gene Ontology (GO)[27], KEGG pathway [28], and Disease Ontology (DO) [29] enrichment analysis for each module. GO annotates genes to biological processes (BP), molecular functions (MF), and cellular components (CC) in a directed acyclic graph structure. We only considered BP terms here. KEGG annotates genes to pathways, and Disease Ontology (DO) annotates genes with human disease associations. To see whether the identified modules show statistically significant, concordant differences between the two diseases, we also did Gene Set Enrichment Analysis (GSEA) [30]. GSEA was also done for the three types of enrichment analysis: GO, KEGG, and DO. We implemented the enrichment analysis with ‘clusterProfiler’ [31]. For all the cases, we let the cutoff be the Benjamini-Hochberg adjusted *p*-value 0.05, and recorded all the enriched terms with *p*-value less than 0.05.

## Results

**Fig. 1:**
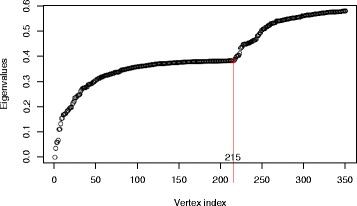
Eigenvalues of matrix *C*

We did module identification in the gene coexpression networks of both breast cancer and ovarian cancer simultaneously. Figure 1 shows the first 350 eigenvalues of the matrix *C*. We chose *M* to be 215. After we clustered the genes into 215 clusters with hierarchical clustering, we removed those with size less than 5 and greater than 800. With the method described, finally, we got 62 modules.

To look at the module structures in both networks, we first computed the average degree (${\bar d}_{BRCA}$, ${\bar d}_{OV}$) and density(*D*_*BRCA*_,*D*_*OV*_) for each module. Besides, we did statistical test for each module to see whether the modules in the two networks have the same connection distribution. We assume the connection between any two vertices is randomly generated following Bernoulli distribution with a given probability. We applied *t*-test to see whether the probability in the two networks is the same. The *p*-values are recorded. For all the 62 modules, after we did all the enrichment analysis, we removed those modules that have no enriched terms. Finally, 43 modules were found to have at least one type of enrichment. We put the module size, the average degree, the density, the *t*-test *p*-value, and the number of enriched terms for GO, KEGG pathway, DO, GSEA in Table 1. For GSEA, we only found GSEA GO enrichment terms.

**Table 1:** Statistics of the identified modules

No.	Size	${\bar d}_{BRCA}$	${\bar d}_{OV}$	D _*BRCA*_	D _*OV*_	*p*-value	N _*GO*_	N _*KEGG*_	N _*DO*_	N _*GSEA*_
1	66	1.36	7.97	0.02	0.12	1.06E-37	2			
2	56	9.79	7.14	0.18	0.13	2.67E-4	1	1		
3	111	3.59	20.29	0.03	0.18	6.88E-160	6	1		
4	6	0.67	1.67	0.13	0.33	0.39	133	1		
5	202	3.65	16.95	0.02	0.08	2.20E-200	53	1		
6	7	3.43	2.57	0.57	0.43	0.54	42		3	
7	92	1.87	2.15	0.02	0.02	0.37	26	5	21	
8	10	0	3.8	0	0.42	3.33E-06	2	1		
9	71	3.38	10.39	0.05	0.15	3.42E-32		2		
10	50	2.28	11.4	0.05	0.23	5.65E-40			1	
11	14	0.43	2.71	0.03	0.21	6.48E-4	22			
12	8	0.5	1.5	0.07	0.21	0.25	9			
13	11	0.36	2	0.04	0.2	0.02	6			
14	25	1.12	5.84	0.05	0.24	1.76E-11	286	27	53	17
15	16	0.88	2.88	0.06	0.19	3.41E-3	4	9	15	
16	29	1.10	4.97	0.04	0.18	5.33E-10		1	3	
17	6	1.33	4	0.27	0.8	0.01	131	7	1	
18	22	1.27	1.36	0.06	0.06	1	16			
19	22	1	2.82	0.05	0.13	2.11E-3	2	2	4	
20	6	2.33	0.67	0.47	0.13	0.11			15	
21	6	2	3.33	0.4	0.67	0.27	15	2	4	
22	13	0.15	2.31	0.01	0.19	6.02E-4	8			
23	40	1.25	6.85	0.03	0.18	3.19E-20	180	8		
24	22	0.64	2.64	0.03	0.13	2.68E-4		1	5	
25	7	0.86	1.71	0.14	0.29	0.45	1			
26	9	0.22	2.44	0.03	0.31	4.42E-3		1		
27	127	3.40	14.87	0.03	0.12	7.67E-109	24			
28	14	0.57	3.71	0.04	0.29	2.72E-05		4	2	
29	14	5.14	5.43	0.40	0.42	0.88	74	2	17	
30	22	3.91	8.82	0.19	0.42	8.08E-08		2		
31	15	0.67	3.73	0.05	0.27	3.03E-05	1	2		
32	7	2.57	0.29	0.43	0.05	0.01	138	2		
33	213	0.94	49.94	0.004	0.24	0	130	3	16	
34	7	0.86	2	0.14	0.33	0.28	40	2	16	
35	12	1.67	4.17	0.15	0.38	5.77E-3	3		1	
36	6	3.33	2.33	0.67	0.47	0.46	38	6		
37	9	2	4	0.25	0.5	0.05	1	1	8	
38	14	2.14	2.86	0.16	0.22	0.45	36		3	
39	6	2	3.33	0.4	0.67	0.27	37			
40	102	4.16	16.35	0.04	0.16	3.08E-91	8			
41	17	1.65	3.88	0.10	0.24	3.89E-3	7			
42	46	2.78	10.04	0.06	0.22	1.68E-25	2			
43	125	10.54	9.28	0.09	0.07	0.02	7			
Total							31	25	18	1

From the *t*-test *p*-values of the modules in both coexpression networks, the structures of 29 modules are significantly different with *p*-values less than 0.05, of which 25 modules have larger densities in OV. Thirty one modules are enriched by GO terms, 25 modules are enriched by KEGG pathways, 18 modules are enriched by DO terms, and one module is enriched by GSEA GO terms. One module (module 14) is enriched by all the four terms. Nine modules (module 7,15,17,19,21,29,33,34,37) are enriched by GO, KEGG, and DO. We checked the details of each module and the enriched terms. All the enrichment results are put in the Additional files 1, 2, 3, 4 and 5.

### GO enrichment analysis

We listed the modules that have an enriched *p*-value less than 10^−5^ in Table 2. Three modules including module 6, 36, and 39 have the same connection distribution in both cancers. These modules have small sizes, and very significant enrichments. Six among the 7 genes in module 6 are involved in the regionalization and pattern specification process. Module 36 is mainly related to glutathione metabolic process and xenobiotic metabolic process. Five of the 6 genes are involved in these processes. Module 39 is mainly related to skeletal system development. Five among 6 genes are involved in this process. In other three modules 5, 14, and 27, the connections in the OV coexpression network are much denser. There are several isolated genes in the BRCA coexpresson network. These three modules are involved in many complex biological processes significantly. One typical example is the enriched term ‘GO:0016259 selenocysteine metabolic process’. There are 89 genes in the background 16655 genes involved in this process. In module 5, 44 among the 195 (overlapping with the background) genes are involved in this process. For the term ‘GO:0006614 SRP-dependent cotranslational protein targeting to membrane’, 45 genes among the 195 genes are involved in the process compared to the background 108 genes of 16655 genes in this process. These show the high correlations among the genes involved in the same process. Compared to OV, the genes involving in the same biological processes in BRCA have much less correlations, which leads to the sparser module structures.

**Table 2:** Enriched GO terms with *p*-value <10^−5^ in all the modules

No.	GO ID	Description	*p*-value
5	GO:0016259	Selenocysteine metabolic process	4.86E-59
	GO:0006614	SRP-dependent cotranslational protein targeting to membrane	2.03E-56
	GO:0006613	Cotranslational protein targeting to membrane	3.84E-56
	GO:0045047	Protein targeting to ER	7.94E-56
	GO:0072599	Establishment of protein localization to endoplasmic reticulum	4.45E-55
	GO:0070972	Protein localization to endoplasmic reticulum	4.45E-55
	GO:0001887	Selenium compound metabolic process	1.37E-53
	GO:0000184	Nuclear-transcribed mRNA catabolic process, nonsense-mediated decay	1.64E-52
	GO:0019080	Viral gene expression	2.59E-47
	GO:0006415	Translational termination	3.29E-47
	GO:0019083	Translational elongation	7.19E-47
	GO:0044033	Multi-organism metabolic process	2.32E-46
	GO:0000956	Nuclear-transcribed mRNA catabolic process	6.16E-44
	GO:0006612	Protein targeting to membrane	6.16E-44
	GO:0006402	mRNA catabolic process	2.23E-42
	GO:0006401	RNA catabolic process	1.09E-39
	GO:0043624	Cellular protein complex disassembly	7.83E-39
	GO:0006413	Translational initiation	1.70E-38
	GO:0043241	Protein complex disassembly	6.44E-38
	GO:0032984	Macromolecular complex disassembly	3.32E-37
	GO:0006575	Cellular modified amino acid metabolic process	3.50E-34
	GO:1901605	Alpha-amino acid metabolic process	1.89E-33
	GO:0090150	Establishment of protein localization to membrane	1.44E-32
	GO:0034655	Nucleobase-containing compound catabolic process	3.53E-32
	GO:0019058	Viral life cycle	1.09E-30
	GO:0044270	Cellular nitrogen compound catabolic process	4.69E-30
	GO:0046700	Heterocycle catabolic process	4.69E-30
	GO:0019439	Aromatic compound catabolic process	1.27E-29
	GO:1901361	Organic cyclic compound catabolic process	2.56E-28
	GO:0072657	Protein localization to membrane	5.23E-28
	GO:0042254	Ribosome biogenesis	3.59E-14
	GO:0022613	Ribonucleoprotein complex biogenesis	5.19E-14
	GO:0042255	Ribosome assembly	1.56E-12
	GO:0042273	Ribosomal large subunit biogenesis	3.61E-12
	GO:0000027	Ribosomal large subunit assembly	9.99E-11
	GO:0022618	Ribonucleoprotein complex assembly	1.23E-09
	GO:0071826	Ribonucleoprotein complex subunit organization	2.74E-09
	GO:0042274	Ribosomal small subunit biogenesis	5.64E-06
6	GO:0009952	Anterior/posterior pattern specification	1.91E-10
	GO:0003002	Regionalization	1.90E-09
	GO:0007389	Pattern specification process	7.21E-09
	GO:0001501	Skeletal system development	1.71E-06
14	GO:0032496	Response to lipopolysaccharide	1.02E-08
	GO:0002237	Response to molecule of bacterial origin	1.02E-08
	GO:0050727	Regulation of inflammatory response	5.43E-08
	GO:1903034	Regulation of response to wounding	8.40E-07
	GO:0030595	Leukocyte chemotaxis	1.24E-06
	GO:0050900	Leukocyte migration	4.99E-06
	GO:0060326	Cell chemotaxis	5.27E-06
27	GO:0044782	Cilium organization	3.90E-10
	GO:0060271	Cilium morphogenesis	7.14E-09
	GO:0042384	Cilium assembly	7.14E-09
	GO:0007018	Microtubule-based movement	1.80E-08
	GO:0010927	Cellular component assembly involved in morphogenesis	7.24E-08
	GO:0030031	Cell projection assembly	3.37E-07
	GO:0042073	Intraciliary transport	4.27E-06
	GO:0098840	Protein transport along microtubule	4.27E-06
36	GO:1901685	Glutathione derivative metabolic process	8.14E-12
	GO:1901687	Glutathione derivative biosynthetic process	8.14E-12
	GO:0006749	Glutathione metabolic process	1.03E-10
	GO:0042537	Benzene-containing compound metabolic process	1.49E-09
	GO:0006805	Xenobiotic metabolic process	2.90E-08
	GO:0071466	Cellular response to xenobiotic stimulus	2.90E-08
	GO:0009410	Response to xenobiotic stimulus	2.90E-08
	GO:0044272	Sulfur compound biosynthetic process	3.74E-08
	GO:0006575	Cellular modified amino acid metabolic process	2.18E-07
	GO:0006790	Sulfur compound metabolic process	5.04E-07
39	GO:0048706	Embryonic skeletal system development	1.28E-08
	GO:0009952	Anterior/posterior pattern specification	7.49E-08
	GO:0048704	Embryonic skeletal system morphogenesis	4.26E-07
	GO:0003002	Regionalization	4.47E-07
	GO:0007389	Pattern specification process	1.51E-06
	GO:0001501	Skeletal system development	1.86E-06
	GO:0048705	Skeletal system morphogenesis	4.37E-06

### KEGG enrichment analysis

The KEGG pathways that enrich the modules having a *p*-value less than 10^−4^ are listed in Table 3. Module 5 is enriched by the pathway ‘hsa03010 Ribosome’. Forty-six out of 130 (overlapping with the background) genes in this module belong to this pathway compared to 154 out of 7274 in the background genes. From the GO enriched terms of this module, it is clear that this module is mainly involved in the translation process. The pathways related to module 14 are mainly related to diseases. ‘IL-17 signaling pathway’ [32], ‘TNF signaling pathway’ [33], ‘Rheumatoid arthritis’ [34], and ‘MAPK signaling pathway’ [35] were shown to have relations with BRCA. For these enriched terms, no existing literatures addressed their associations with OV to the best of our knowledge. Module 23 is also related to cancers. Eight genes in this module belong to ‘Pathways in cancer’. It is also enriched by ‘breast cancer’ with a *p*-value 0.049, with 3 genes associated with breast cancer in this module. This module has no enrichments related to OV. The pathway ‘hsa05418 Chemical carcinogenesis’ that enriches module 36 is also related to cancers. Chemical carcinogens may contribute significantly to the causation of a sizable fraction, perhaps a majority, of human cancers [36].

**Table 3:** Enriched KEGG terms with *p*-value <10^−4^ in all the modules

No.	KEGG ID	Description	*p*-value
5	hsa03010	Ribosome	1.02E-43
14	hsa04668	TNF signaling pathway	3.80E-09
	hsa04657	IL-17 signaling pathway	1.97E-06
	hsa05323	Rheumatoid arthritis	4.57E-05
	hsa04380	Osteoclast differentiation	1.96E-04
	hsa04010	MAPK signaling pathway	3.00E-04
23	hsa05217	Basal cell carcinoma	2.59E-05
	hsa05200	Pathways in cancer	9.26E-05
33	hsa04610	Complement and coagulation cascades	2.68E-06
36	hsa00480	Glutathione metabolism	1.12E-10
	hsa00982	Drug metabolism-cytochrome P450	1.43E-10
	hsa01524	Platinum drug resistance	1.43E-10
	hsa00980	Metabolism of xenobiotics by cytochrome P450	1.43E-10
	hsa05204	Chemical carcinogenesis	1.93E-10
	hsa05418	Fluid shear stress and atherosclerosis	2.37E-09

### DO enrichment analysis

Table 4 lists the DO enriched terms in all the modules with *p*-value less than 0.01, and one term that is related to OV. Module 14 is enriched by BRCA, with 7 among 20 (overlapping with the background) genes in this module associated with BRCA. Module 38 is enriched by OV with 4 among 11 (overlapping with the background) genes in this module being associated with OV. There are no OV related enriched terms in module 14, and no BRCA related terms in module 38. This is mainly due to the different known genes associated with the two cancers. We note that module 34 is also enriched by the female organ cancer.

**Table 4:** Enriched DO terms with *p*-value <0.01 in all the modules

No.	DO ID	Description	*p*-value
14	DOID:3770	Pulmonary fibrosis	7.40E-04
	DOID:1602	Lymphadenitis	7.40E-04
	DOID:9942	Lymph node disease	7.40E-04
	DOID:1936	Atherosclerosis	7.40E-04
	DOID:1036	Chronic leukemia	7.40E-04
	DOID:2348	Arteriosclerotic cardiovascular disease	7.40E-04
	DOID:2349	Arteriosclerosis	7.79E-04
	DOID:3459	Breast carcinoma	1.10E-03
	DOID:3082	Interstitial lung disease	1.94E-03
	DOID:0070004	Myeloma	3.21E-03
	DOID:75	Lymphatic system disease	3.21E-03
	DOID:4960	Bone marrow cancer	3.21E-03
	DOID:865	Vasculitis	5.68E-03
	DOID:13378	Kawasaki disease	7.82E-03
	DOID:9538	Multiple myeloma	9.27E-03
15	DOID:2730	Epidermolysis bullosa	3.27E-04
	DOID:2731	Vesiculobullous skin disease	3.27E-04
	DOID:299	Adenocarcinoma	6.72E-03
	DOID:4766	Embryoma	9.26E-03
	DOID:688	Embryonal cancer	9.71E-03
17	DOID:700	Mitochondrial metabolism disease	1.77E-03
33	DOID:1882	Atrial heart septal defect	2.16E-03
	DOID:9477	Pulmonary embolism	2.16E-03
	DOID:1681	Heart septal defect	8.06E-03
	DOID:1682	Congenital heart disease	8.06E-03
	DOID:2214	Inherited blood coagulation disease	8.06E-03
	DOID:780	Placenta disease	8.13E-03
34	DOID:2893	Cervix carcinoma	1.62E-03
	DOID:4362	Cervical cancer	1.62E-03
	DOID:120	Female reproductive organ cancer	4.60E-03
37	DOID:10652	Alzheimer’s disease	8.20E-03
	DOID:680	Tauopathy	8.20E-03
38	DOID:3113	Papillary carcinoma	3.66E-03
	DOID:2394	Ovarian cancer	4.14E-02

From all the above analysis, we found that most modules are the general modules that may not be associated with BRCA and OV. The validated enriched terms related to BRCA are much more than that of OV. One reason may be there are more researches conducted on BRCA. Among all the enriched modules, module 14 is enriched by BRCA, and module 38 is enriched by OV. Module 36 is not enriched by these two diseases, but the enriched terms are related to cancer treatment. Among these three modules, the structure of module 14 is shown to be different with a *p*-value 1.76E-11 in these two cancers. In the following, we give some details of these three modules.

### Enrichment analysis for module 14

**Fig. 2:**
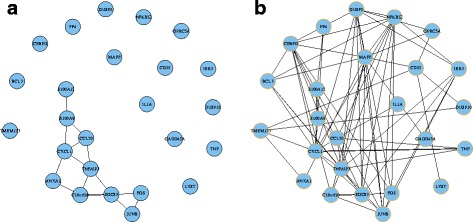
Network structure of module 14. **a** BRCA; **b** OV

**Fig. 3:**
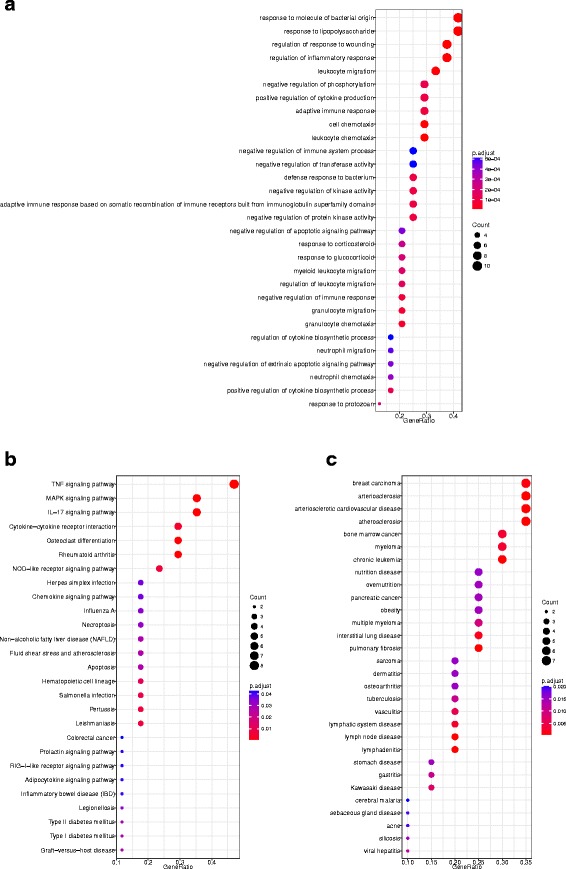
Enrichment results for module 14. **a** GO, 30 terms with minimal *p*-values; **b** KEGG, all enriched 27 terms; **c** DO, 30 terms with minimal *p*-values

**Fig. 4:**
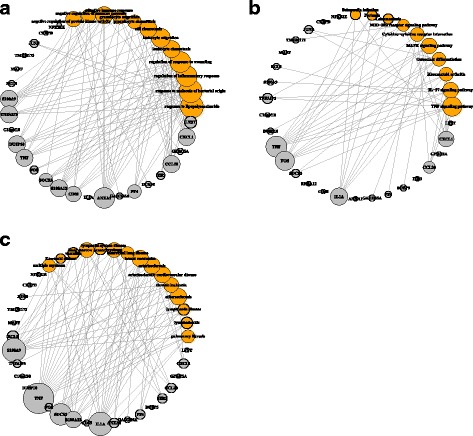
Enrichment results for module 14. **a** GO terms with *p*-value <10^−4^; **b** KEGG terms with *p*-value <0.01; **c** DO terms with *p*-value <0.01

Module 14 consists of 25 genes. The module structure in both networks is significantly different with a *p*-value 1.76E-11. Figure 2 shows the module structures. It is much denser in OV compared to that in BRCA. Figure 3 shows the dotplot of GO, KEGG, DO enrichment results. For GO and DO, we plotted the first 30 enriched terms with the smallest *p*-values. We plotted all the 27 enriched terms for KEGG. Figure 4 shows the associations between the genes and the enriched terms. We selected the most enriched 12 GO terms with *p*-value less than 10^−4^, 10 KEGG terms with *p*-value less than 0.01, and 15 DO terms with *p*-value less than 0.01.

The enriched GO terms are mainly related to different responses, such at inflammatory response, response to molecule of bacterial origin, responses to wounding, immune response, etc.. Several responses have been shown to be associated with cancers [37, 38]. For example, the inflammation as the seventh hallmark of cancer plays important roles in cancer development. Inflammatory cells may facilitate angiogenesis and promote the growth, invasion, and metastasis of tumor cells, which may change the genetic instability in cancer cells. Controlling the regulation of inflammatory response has a potential in both prevention and treatment of cancer [37]. There are 9 genes included in this module associated with the enriched term ‘regulation of inflammatory response’, which achieves a *p*-value of 5.43E-08. This module also includes 7 genes that are associated with the enriched term ‘adaptive immune response’, which achieves a *p*-value of 8.78E-05. The immune response can result in the proliferation of antigen-specific lymphocytes. When antibodies and T-cell receptors are expressed and up-regulated, immunity is acquired. Then the immune systems will initiate antigenic responses against carcinomas. A new approach to the treatment of cancer is immunotherapy, which aims to up-regulate the immune system in order that it may better control carcinogenesis [38]. Figure 4a shows the relations between the 12 GO terms with *p*-value less than 10^−4^ and the 25 genes in this module. The genes ‘CXCL1’, ‘CCL20’, ‘ANXA1’, ‘S100A9’, ‘S100A12’, ‘TNF’, ‘DUSP10’, ‘TNFAIP2’ are included in more than 6 enriched terms. There are 10 genes associated with the most enriched terms ‘response to lipopolysaccharide’ and ‘response to molecule of bacterial origin’. For these two terms, we have not found the related literature that addresses their relations to cancers.

In the KEGG enriched terms, ‘TNF signaling pathway’ [33],‘Rheumatoid arthritis’ [34], ‘MAPK signaling pathway’ [35], and ‘IL-17 signaling pathway’ [32] have shown to be associated with BRCA. TNF is a major inflammatory cytokine shown to be highly expressed in breast carcinomas. It induces a wide range of intracellular signal pathways including apoptosis and cell survival as well as inflammation and immunity [33]. ‘MARK signaling pathway’ is involved in various cellular functions, including cell proliferation, differentiation and migration. Research on signaling pathway switch in breast cancer shows that in a large proportion of breast cancer, MARK signaling pathway is repressed, while another important pathway is activated. This mechanism may have impacts on the balance between self-renewal, proliferation, and differentiation of the tumor-initiating cells [35]. IL-17 plays crucial roles in both acute and chronic inflammatory responses. It is shown to have a direct association with breast cancer invasion in human breast tumors. IL-17 directly induced breast cancer cell invasion. There should be a potential mechanism for breast cancer invasion and tumor progression [32]. ‘Rheumatoid arthritis’ is mainly related to immune systems. Research shows that the risk of breast cancer is increased in non-Caucasians patients with rheumatoid arthritis while it decreased in Caucasian population [34]. Figure 4b shows the associations between the enriched KEGG terms and the genes. Four genes including ‘CXCL1’, ‘TNF’, ‘FOS’, and ‘IL1A’ connect to at least 6 of the 10 terms. There are at least 6 genes associated with ‘TNF signaling pathway’, ‘MAPK signaling pathway’, and ‘IL-17 signaling pathway’. For these enriched pathways, we have not found their associations with OV.

In the DO enriched terms, ‘breast carcinoma’ reaches the *p*-value 0.001. Seven genes including ‘S100A9’, ‘TNF’, ‘SOC53’, ‘CD55’, ‘IL1A’, ‘ANXA1’, ‘GADD45A’ among the 25 genes in this module are associated with BRCA. Several other diseases also enrich module 14, including arteriosclerosis disease, nutrition disease, etc.. However, OV is not on this list. Figure 4c shows the associations between the genes and the diseases. ‘S100A9’, ‘TNF’, ‘SOCS3’, and ‘IL1A’ connect to at least 10 diseases among the 15 enriched diseases.

**Fig. 5:**
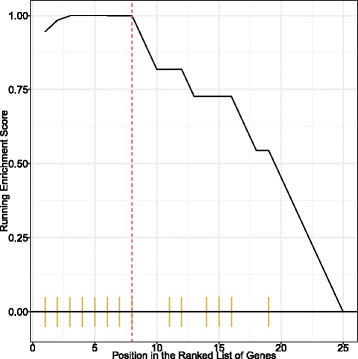
GSEA GO enrichment for module 14

In the GSEA study, we ordered the genes according to the *t*-test *p*-value between the two diseases and did the analysis. Finally, 17 GO terms enrich this module. Table 5 shows the enriched terms. The 8 sequential genes having the largest *t*-test *p*-values are all in the enriched biological processes. They are ‘TNFAIP3’, ‘S100A9’, ‘BCL3’, ‘MAFF’, ‘TMEM173’, ‘JUNB’, ‘CEBPD’, ‘NFKBIZ’. Figure 5 shows the patterns for the running enrichment score. All the enriched terms have a similar pattern for these 8 genes.

**Table 5:** Enriched GO terms with GSEA for module 14

GO ID	Description	Set Size	*p*-value
GO:0010467	Gene expression	14	0.04
GO:0010468	Regulation of gene expression	14	0.04
GO:0034645	Cellular macromolecule biosynthetic process	14	0.04
GO:0006351	Transcription, DNA-templated	13	0.04
GO:0006355	Regulation of transcription, DNA-templated	13	0.04
GO:0016070	RNA metabolic process	13	0.04
GO:0018130	Heterocycle biosynthetic process	13	0.04
GO:0019438	Aromatic compound biosynthetic process	13	0.04
GO:0032774	RNA biosynthetic process	13	0.04
GO:0034654	Nucleobase-containing compound biosynthetic process	13	0.04
GO:0044271	Cellular nitrogen compound biosynthetic process	13	0.04
GO:0051252	Regulation of RNA metabolic process	13	0.04
GO:0097659	Nucleic acid-templated transcription	13	0.04
GO:1901362	Organic cyclic compound biosynthetic process	13	0.04
GO:1903506	Regulation of nucleic acid-templated transcription	13	0.04
GO:2000112	Regulation of cellular macromolecule biosynthetic process	13	0.04
GO:2001141	Regulation of RNA biosynthetic process	13	0.04

By comparison of this module structure between BRCA and OV, we found module 14 is closely related to BRCA from the above enrichment analysis. From the gene coexpression network of BRCA (Fig. 2), we see some breast cancer associated genes are isolated, such as ‘TNF’, ‘CD55’, ‘IL1A’. In OV network, these genes are highly correlated and clustered into one module. This may show that due to the tumor, the correlations of some cancer related genes decrease in BRCA.

### Enrichment analysis for module 36

This module includes 6 densely connected genes in both diseases, which has a *p*-value 0.46 in *t*-test for the structure difference. Of the 6 genes, 5 genes GSTM1, GSTM2, GSTM3, GSTM4, GSTM5 encode the glutathione S-transferase that belongs to the mu class. These genes function in the detoxification of electrophilic compounds such as carcinogens, therapeutic drugs, environmental toxins and products of oxidative stress, by conjugation with glutathione. Thus this module is enriched by the related biological processes such as: ‘glutathione derivative metabolic process’, ‘xenobiotic metabolic process’, ‘sulfur compound biosynthetic process’, etc.. It is also enriched by the related pathways such as ‘glutathione metabolism’, ‘drug metabolism-cytochrome P450’, ‘chemical carcinogenesis’, and so on. Another gene is BCAR3, which is associated with estrogen resistance and breast cancer. It is translated to the breast cancer anti-estrogen resistance protein 3. Although this module is not enriched by BRCA and OV in DO significantly, it is related to the treatment of breast cancer [39].

### Enrichment analysis for module 38

**Fig. 6:**
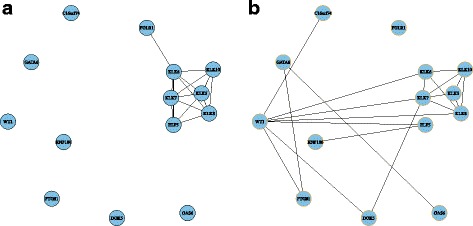
Network structure of module 38. **a** BRCA; **b** OV

Module 38 includes 11 genes. Figure 6 shows the module structure in both cancers. The connection probability in these two networks is statistically the same with *t*-test, although the detailed connections are different. This module is enriched by 36 GO terms with adjusted *p*-value less than 0.05. The gene number involved in the related biological processes is at most 4. It is enriched by 3 diseases including ovarian cancer. Four of the 14 genes are associated with OV using DO enrichment. One typical gene is ‘WT1’, which connects to several other genes in the OV network, while it has no connections in the BRCA network. This gene is necessary for the development of the ovaries in females, and thus is associated with ovarian cancer. The ‘WT1’ protein has been found to bind a host of cellular factors such as p53. It has been ranked as the No.1 target for cancer immunotherapy by the National Cancer Institute. However, it has no associations with BRCA to the best of our knowledge. A densely connected subnetwork in this module is Kallikrein-related peptidases (KLK5, KLK6, KLK7, KLK8, KLK10). This gene family can be taken as the novel cancer biomarkers as shown in [40]. The potential of KLKs as diagnostic, prognostic, and treatment monitoring biomarkers for many types of malignancies has been extensively investigated including breast cancer and ovarian cancer. Overall, this module is closely related to cancers including breast cancer and ovarian cancer, and is more associated with ovarian cancer.

## Discussion and conclusion

Breast cancer and ovarian cancer are important causes of death for women. Both of them are harmone driven and are known to have some common susceptible genes such as BRCA1 and BRCA2. Several published works have studied both cancers together with the assumption that they have the same etiologies. Coexpression network modules for both breast cancer and ovarian cancer have been studied separately by several researchers. However, there are no comparisions between the coexpression network modules between breast cancer and ovarian cancer. By comparing the modules in both cancers, we aim at finding more relations between both cancers including both the similarities and the differences.

In this work, we compared the coexpression network modules of both cancers by simultaneously identifying the modules of both cancers. By projecting the genes into a lower space representation, we did hierarchical clustering of all the genes with complete linkage of spearman distance to get the modules. 43 modules were identified to be enriched by at least one of GO, KEGG pathway, and DO terms. In most of these modules, density in OV network is larger than that in BRCA network. There are 31, 25 and 18 modules enriched by GO, KEGG pathway, and DO terms, respectively. By checking the details of each enriched term, we found that one module (module 14) is enriched by GO, KEGG and DO terms related to breast cancer. Among all GO and KEGG enriched terms, several have been validated. And there are 7 genes associated with breast cancer in this module. However, there is no enrichment information related to ovarian cancer. Another module 38, which is enriched by ovarian cancer in DO, is closely related to several cancers including breast cancer and ovarian cancer, and is more associated with ovarian cancer. From the analysis of the genes in module 36, we found that it is related to the treatment of breast cancer, although it is not enriched by breast cancer terms in DO.

Comparison of the identified modules shows the differences of breast cancer and ovarian cancer on the module level. Different modules are enriched by the two cancers significantly. It also shows some common properties of both cancers such as the KLKs family in module 36. More importantly, by simultaneous clustering of both cancers, the potential cancer related modules can be identified. For example, module 14 is significantly enriched by breast cancer related terms, but much connection information is borrowed from the ovarian cancer network. This may imply the associations between this module and breast cancer.

## Additional files

Additional file 1 The identified modules. We listed all the identified modules. The second column in each sheet lists the gene names. (XLSX 337 kb)

Additional file 2 Statistics of the modules. The module size, average degree, density, number of enriched GO, KEGG, DO terms are listed. The module names are the sheet name in Additional file 1. There is a map between the module names and the module number in the paper. (CSV 5 kb)

Additional file 3 GO enrichment results. This file lists all the GO-BP enriched terms for each module. ‘geneID’ is the Entrez gene id. (XLSX 193 kb)

Additional file 4 KEGG enrichment results. This file lists all the KEGG enriched terms for each module. ‘geneID’ is the Entrez gene id. (XLSX 59 kb)

Additional file 5 DO enrichment results. This file lists all the DO enriched terms for each module. ‘geneID’ is the Entrez gene id. (XLSX 54 kb)
